# Fanconi anemia: young patients at high risk for squamous cell carcinoma

**DOI:** 10.1186/s40348-014-0009-8

**Published:** 2014-11-01

**Authors:** Eunike Velleuer, Ralf Dietrich

**Affiliations:** Clinic for Pediatric Oncology, Hematology and Clinical Immunology, Children’s Hospital, University Hospital of Düsseldorf, Moorenstr. 5, 40225 Düsseldorf, Germany; Deutsche Fanconi-Anämie-Hilfe e.V., Böckenweg 4, 59427 Unna-Siddinghausen, Germany

**Keywords:** Fanconi anemia, Young patients, Squamous cell carcinoma, Bone marrow transplant

## Abstract

**Background:**

Fanconi anemia is one of the best studied inherited cancer-prone diseases. Greatly improved protocols for hematopoietic stem cell transplantation increasingly save the lives of these young patients. However, in both transplanted and not transplanted patients, the emergence of aggressive squamous cell carcinoma represents a major medical challenge.

**Conclusions:**

This mini review summarizes current knowledge about the pathogenesis of squamous cell carcinoma (SCC) in the special context of Fanconi anemia.

## Introduction

Fanconi anemia is a rare inherited condition with an incidence of approximately 1 in 200,000 births and a median life expectancy in the mid-1920s. With the exception of the X-chromosomal FANCB gene, biallelic mutations in any of 15 FANC genes cause the recessive multisystem disorder with highly variable clinical presentation. In addition to progressive hematological anomalies, 70% of FA patients display congenital anomalies (including short stature, radial ray and renal defects, café au lait spots, microphthalmia, and hearing difficulties). From a clinical point of view, it is important to realize that 30% of FA patients lack physical impairments although they share the greatly increased risk of bone marrow failure, MDS, AML, and squamous cell carcinomas (SCC). The clinical suspicion of FA is confirmed by the demonstration of increased spontaneous and induced chromosome breakage, excessive accumulation of cells in the G2 phase of the cell cycle, or targeted DNA sequencing. Symptomatic treatment consists of blood product substitution and androgens, but recent improvements render related or unrelated donor HSCT the treatment of choice. Although curative with respect to bone marrow failure, HSCT does not prevent the emergence of SCC.

In a competing risk analysis, the estimated risk for the development of any solid tumor by age 45 amounted to 75% [1,2]. The median age of onset of SCC in FA is around 33 years, thus preceding that of the general population by 30 to 40 years. Compared to the general population, the overall risk is increased by 500- to 700-fold for head and neck SCC (HNSCC), 2,000-fold for esophageal cancer, and 4,000-fold for vulvar cancer [3]. Most of the published and best studied SCC in FA patients are located in the oral cavity [4].

## Clinical dilemma of HNSCC in FA

Compared to the non-FA population, two thirds of the HNSCC in FA patients are located within the oral cavity, most frequently at the tongue margins and the gingival areas. In strong contrast to the non-FA (and mostly male) population, these tumors arise in both male and female FA patients without a prior history of excessive tobacco and alcohol use [4]. Compared to untransplanted FA patients, a history of HSCT (and a history of GvHD in particular) increases the risk of HNSSC by 4.4-fold. In transplanted patients, onset occurs approximately 10 years earlier [5]. Sixty-three percent of patients present with late stage and multiple tumors developing either synchronously or metachronously [4].

Even if a patient presents at an early stage, there are frequent relapses. The highly aggressive nature of the tumors is reflected by poor survival, amounting to less than 2 years [6]. Among published cases, 20% of FA-related HNSCC patients presented with these tumors as the first sign of FA. To our knowledge, there is no report in the FA literature validating the appearance of potentially malignant disorders such as lichen planus, erythroplakia, and leukoplakia. In our own experience with more than 500 patients, these prodromal signs appear to be quite frequent.

**Figure 1:**
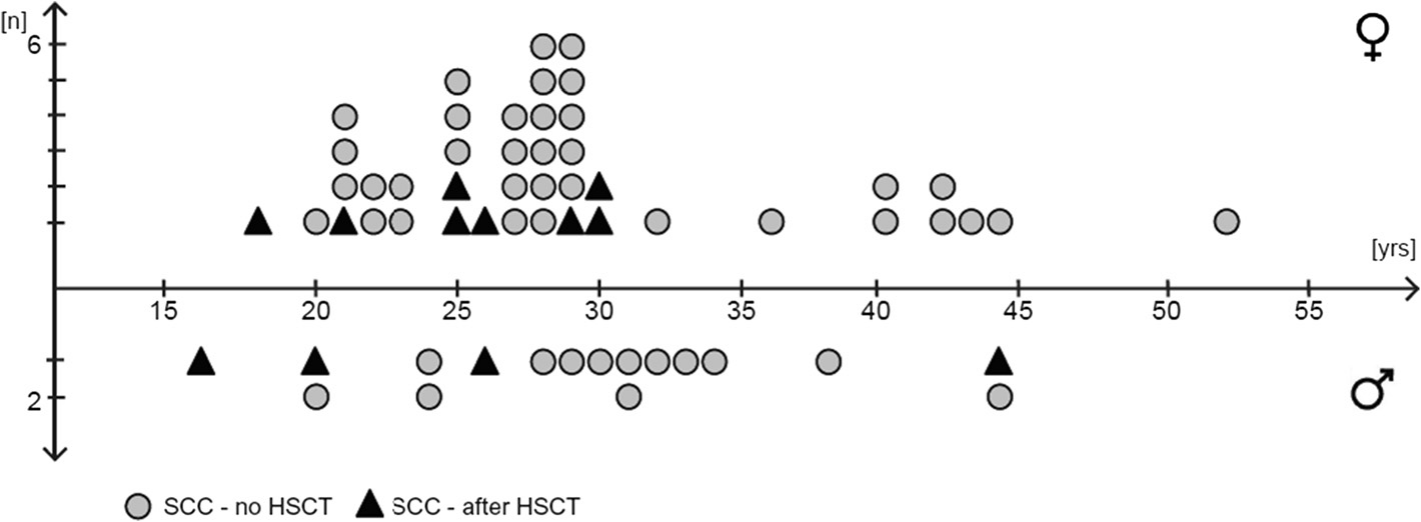
**Age of onset of SCC in FA patients with and without HSCT.** Forty-three of 83 female FA patients (51.8%) and 17 of 46 male FA patients (37.0%) developed SCC (average age at SCC diagnosis for 48 FA patients without HSCT 30.0 years, for 12 FA patients after HSCT 25.8 years). Data summarized from Hohnbaum (see text).

There is a limited number of reports describing the epidemiology and natural history of FA-related SCC [2]. One of the most recent and largest collections of young adult and adult FA patients affected by such tumors was published by Grit Hohnbaum in 2008 [http://d-nb.info/997016965]. As summarized in Figure 1, Hohnbaum compiled data of 129 FA patients 20 years and older (comprising 42 case reports from the literature (1964 to 2008), 36 case histories from newsletters of the German and US FA family support groups (www.fanconi.de and www.fanconi.org), and 51 FA patients with cell cycle studies performed at the Institute of Human Genetics of the University of Wurzburg, Germany). Overall, 60 of these 129 patients (48 no HSCT, 12 after HSCT) developed SCC during their lifetime. The average age at SCC diagnosis for transplanted patients was 25.8 years (range 16 to 44 years) and for non-transplanted patients 30.0 years (range 20 to 52 years).

Due to intrinsic genetic instability and hypersensitivity towards DNA crosslinking agents in FA, treatment options are limited, excluding standard protocols involving cisplatinum. Surgery is the preferred option, but given the frequently advanced tumor stage, additional treatments, such as radiotherapy, are required. Given the pronounced sensitivity of FA cells towards oxygen tension, it remains to be seen whether hypoxic modification of the radiation protocol may be of benefit for FA patients. Under hypoxic (i.e., physiological) cell culture conditions, FA-derived fibroblasts have been shown to tolerate ionizing radiation as well as control cultures derived from unaffected donors [7]. However, there are contradictory reports on *in vivo* radiosensitivity of FA patients, with those tolerating up to 70 Gy without major problems, whereas other patients suffer from severe side effects even at low-dose exposures [8]. Patients with a favorable response to radiation may represent mosaics (carrying somatic reversions of one of their defective FA alleles in their blood cells). They may also be carriers of the so-called ‘hypomorphic’ mutations (presence of residual and functional FA protein in contrast to ‘null’ mutations with complete absence of functional FA protein). Whether transplanted patients tolerate radiation therapy during SCC treatment better than not transplanted patients requires further study. To date, the use of alternative treatment options such as epidermal growth factor inhibitors (e.g., cetuximab) is slowly increasing in FA patients [8].

## Cellular and molecular basis of HNSSC in FA

Whereas the increased susceptibility of FA patients to early-onset SCC (largely in the absence of known external risk factors) has been noted for decades, the question of why the FA genotype is particularly prone to the development of these particular types of tumors at quite specific anatomical sites remains unanswered. Since many of the known human genetic instability syndromes (including ataxia telangiectasia, Bloom syndrome, and Werner syndrome) share the increased risk of early-onset neoplasia with FA, there is little doubt that genetic instability *per se* and intrinsically promotes carcinogenesis. Joenje et al. have shown the rate of chromosomal breakage in FA cells to be a clear function of the oxygen concentration in the culture environment [9]. By growing FA cells under hypoxic culture conditions (5% *v*/*v* oxygen), chromosomal breakage can be all but eliminated. There is also *in vivo* evidence for increased oxidative stress in FA, suggesting an imbalance in the various cellular redox systems [10]. Since reactive oxygen species are known to damage DNA, they are likely to contribute to carcinogenesis, most plausibly in the absence of functional DNA repair pathways. In this context, it seems intriguing that the preferred anatomical sites at which SCC develop in FA involve areas exposed to atmospheric oxygen (20% *v*/*v* oxygen). However, clear evidence for a protective role of the FA family of genes against oxygen toxicity is still lacking.

**Figure 2:**
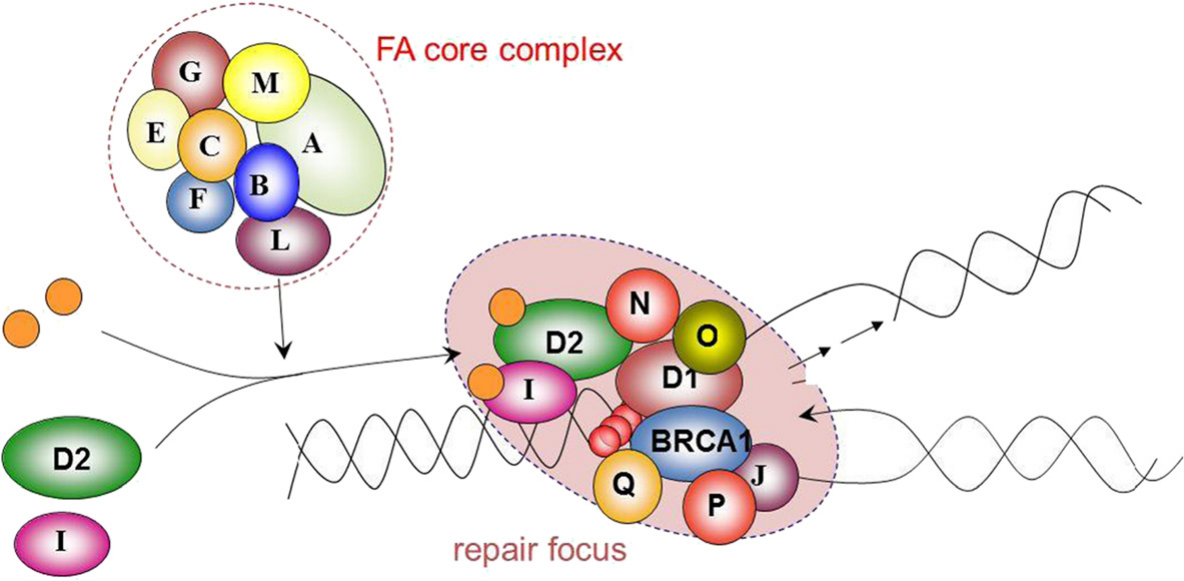
**Simplified model of the Fanconi anemia pathway.** Activation of FANCD2 and FANCI by the FA core complex via monoubiquitination (orange circles) regulates downstream genes involved in recombination repair of DNA crosslinks.

Our current understanding of the role of the FA proteins reflects their pivotal function in the surveillance and maintenance of genomic integrity. As pointed out by Romick-Rosendale and co-workers in a recent review [11], the frequent emergence of SCC in FA must be seen in the context of defective DNA repair compromising genomic integrity. Owing to the rapid pace of FA gene discovery during recent years, the eminent role of FA proteins in recombinational types of DNA repair has emerged (cf. Figure 2). Briefly, in response to crosslink type of DNA damage and stalled replication forks, eight of the known FA proteins assemble into the so-called FA core complex which leads to the activation (via monoubiquitination) of the FANCD2 and FANCI proteins and, in turn, to the activation of a number of ‘downstream’ proteins instrumental in DNA repair. Interestingly, monoallelic mutations in some of the downstream proteins are known to confer a high risk of breast cancer (e.g., FANCD1 = BRCA2, FANCN = PALPB2, FANCJ = BRIP1, FANCO = RAD51C). Again, these observations emphasize the intimate connection between inadequate or failing DNA maintenance and the emergence of neoplasia. Persistent or misrepaired DNA damage results in cell cycle arrest, apoptosis, and chromosomal instability and, ultimately, in the complex patterns of somatic mutations and epimutations which characterize malignant cell populations.

## Role of FA proteins in sporadic cancers

Contrary to expectation, most studies of sporadic cancers have shown the presence of intact and functioning FA genes. This implies that the majority of tumor cells, at least during the initial stages of tumor growth, require the presence of intact FA genes. A frequently reported presumptive methylation defect (and thereby loss of function) of the FANCF gene in many different types of cancers could not be confirmed with improved technology [12]. Molecular alterations of a FA gene (FANCC) have been observed in occasional sporadic SCC tumors, being associated with higher recurrence rate and shorter disease-free survival [13]. Given the highly complex pattern of genetic alterations which, in advanced tumors, may also involve members of the FA family of genes, these limited observations cannot answer the question whether alterations involving FA genes in a given tumor act as driver or passenger mutations. The time- and stage-dependent accumulation of genetic alterations, as evidenced by loss of heterozygosity, suggests that a multistep process characterizes the natural history of sporadic SCC [14]. Because of the obviously intrinsic and highly aggressive nature of SCC in FA patients, it was initially hoped to find differences in the genetic makeup between FA and non-FA SCC cells. However, a study by van Zeeburg et al. [15] involving 21 FA-derived SCC showed that the patterns of allelic loss were similar to those observed in sporadic SCC.

## Extrinsic factors which may promote HNSCC development in FA

Excessive and long-term tobacco and alcohol consumption have been clearly established as external factors in the emergence of sporadic head and neck cancers, with tobacco abuse being by far the major culprit in some studies [16]. With regard to sporadic HNSCC, other proven or suggestive associations have been described with human papilloma virus (HPV) infection, with changes in the oral microbiome, and with bad oral hygiene culminating in chronic oral infections [11].

The important role of HPV is highlighted by the fact that virus types 16 and 18 have been found in 25% of sporadic HNSCC. Molecular studies have shown that an intact FA pathway is necessary to restrict the HPV life cycle. *Vice versa*, loss of a functional FA pathway is associated with accumulation of E7, causing increased epithelial proliferation and basal cell layer expansion in HPV-positive epidermis [11]. There are important differences between HPV-associated sporadic tumors and tumors arising in FA patients: most HPV-positive sporadic tumors are located in the oropharynx, whereas most FA-associated tumors are located in the oral cavity. A similar difference holds for gynecological tumors, with HPV-related tumors affecting the cervix but FA-related tumors affecting the vulva and anus. It therefore came as a surprise that a US study of Kutler et al. [17] reported HPV in 84% of 25 SCC derived from 24 FA patients. Likewise, 6 of 7 cancers of the vulva and 15 of 18 HNSCC were found to be HPV positive. A European study by van Zeeburg et al. was unable to confirm these high rates of HPV in FA [15]. The European authors detected HPV in only 2 of 21 FA-related SCC (10%), with both positive cases involving the anogenital regions. All 16 FA-derived HNSSCs were negative. Technical and/or geographic/ethnic differences between the two studies were extensively discussed, but the controversy could not be resolved. Most recently, Alter and co-workers published a smaller case series involving nine tumors derived from FA patients in the US [18]. In agreement with the van Zeeburg study, only a single of the tumors was HPV positive, and the single HPV-positive tumor originated from the anogenital area. Collectively, the more recent studies suggest that HPV infection may play a more prominent role in sporadic compared to FA-related SCC. In addition, HPV-positive sporadic tumors seem to have a better prognosis than HPV-negative tumors. As mentioned before, SCC that emerge on a FA genetic background are unusually aggressive, are preferentially located in the oral cavity rather than the oropharynx, and are more difficult to treat because of the vulnerability of the FA genotype towards crosslinking agents such as cisplatinum. Notwithstanding these differences between sporadic and FA-related SCC (implying differences in the pathogenesis of the respective tumors), vaccination against HPV is generally recommended for prepupertal FA patients of both sexes.

## Environmental carcinogens

In contrast to the proven role of tobacco and alcohol use in sporadic SCC, there is anecdotal evidence at best for occasional smoking and/or drinking in young adult FA patients. In a small study, there was no evidence for excessive tobacco or alcohol use in FA patients who develop SCC [4]. Larger epidemiological studies are lacking.

Our experience with more than 500 interviewed FA patients suggests that young adult FA patients tend to consume alcohol in a social manner, quite comparable to the general population of the respective countries and cultures. The social use of tobacco is clearly less common.

The carcinogenic effects of ethanol have only recently been clarified. They appear to be mediated via acetaldehyde, the first product of alcohol metabolism. On the basis of these findings, the IARC working group rated acetaldehyde derived from alcohol intake as a group I carcinogen. It was further discovered that acetaldehydes generate DNA lesions, most notably DNA interstrand crosslinks, which lead to activation of the FA pathway via monoubiquitination of FANCD2. According to these studies, a functional FA pathway is required for the proper elimination of DNA damage induced by acetaldehyde. In addition to their obvious relevance for the pathogenesis of the fetal alcohol syndrome, these findings might also be relevant in the context of FA. For example, a variety of aldehydes occur as natural by-products of intermediary metabolism. Other moieties of aldehydes, such as formaldehydes, are ubiquitously found in the environment and may interfere with intrauterine and postnatal development in a dose-dependent manner.

Murine transgenic models of FA rarely mimic all features of the FA phenotype and thus are of limited use. However, Langevin et al. recently succeeded in creating double knockout mice in which both the FANCD2 alleles and the alleles coding for alcohol dehydrogenase (Aldh2) were inactivated (FancD2−/− plus Aldh2−/−) [19]. They found that only Aldh2+/− mothers were able to give birth to living FancD2−/− and Aldh2−/− offspring whereas Aldh−/− mothers had no life offspring. The authors suggest that only the mothers with at least one functioning Aldh2 allele (Aldh2+/−) were able to detoxify aldehydes generated in their offspring via the placenta. Most importantly, the surviving double knockout (FancD2−/−Aldh2−/−) mice developed a typical FA phenotype with various malformations, progressive bone marrow failure, and death due to leukemia. The authors went a step further and exposed the pregnant Aldh+/− mice to a moderate dose of ethanol. Most of their embryos died and the few survivors showed severe malformations. A logical further step was to study how FancD2−/−Aldh2−/− mice themselves react towards exposure to ethanol in the drinking water. Most of the mice succumbed to rapid bone marrow failure, emphasizing the lack of protection towards the adverse effects of ethanol in the absence of FANCD2 function. What about humans? A recent study by Hira et al. [20] found accelerated bone marrow failure in FA patient carriers of dominant-negative ALDH2 alleles, confirming the adverse effects of ethanol metabolism in the absence of a functioning FA pathway.

## Oral health and the role of the microbiome

In addition to the main carcinogens tobacco and alcohol, poor oral hygiene and negligent dental care have been identified as possible risk factors for sporadic HNSCC. These associations are thought to result from a shift in the bacterial flora accompanied by a potentially pathogenic inflammatory response setting the stage for chronic inflammation.

In recent years, refined technologies such as 16S rRNA analysis have facilitated whole-microbiome analysis. Several such studies revealed differences in the composition of the microbiota among tumor and non-tumor samples. Whether the presence of different bacteria and/or the disruption of the normal microbiome spectrum within the mucosa contributes to carcinogenesis is worthy of further investigation, including the HNSCC-prone population of FA patients. It also seems worth to follow up on a recent study suggesting that smoking may increase the ability of the microbiome for the production of acetaldehydes [21]. According to this study, tobacco smoke might facilitate the selection of microbes that are capable of a high rate of acetaldehyde metabolism and thus would be more tolerant to these compounds in heavy smokers. It should also be mentioned that xylitol reduces the amount of acetaldehydes produced by *Candida albicans* after ethanol exposure, an observation that might bear potential therapeutic and preventive implications [22]. The ability of bacteria and fungi to produce acetaldehydes highlights the importance of good oral hygiene especially in FA patients.

## Summary

FA patients are at highly increased risk for early-onset squamous cell carcinomas at specific locations (oral cavity and anogenital area), regardless of whether they have been transplanted or not. Reactive oxygen species, DNA crosslinking agents, and aldehydes have been identified as endogenous and exogenous sources of DNA damage which, if un- or misrepaired, contributes to carcinogenesis. Animal experiments have provided evidence for the protective role of the FA pathway in the detoxification of aldehyde compounds. It is strongly recommended that FA patients avoid exogenous sources of aldehydes. Even in the absence of a ‘typical FA phenotype,’ FA must be excluded by laboratory testing in patients presenting with SCC at an early age and without a history of excessive tobacco and alcohol use. Standard treatment with crosslinking agents such as cisplatinum would be fatal in unrecognized FA patients. This includes the so-called ‘mosaic’ patients presenting with normal blood counts and a negative chromosomal breakage or cell cycle test. New therapeutic options like hypoxia-modified radiation or epidermal growth factor inhibitors might improve the poor prognosis of FA patients suffering from advanced SCC. Most importantly, noninvasive screening options, including frequent self-examination and inspection by qualified care givers, should lead to early detection, thus bearing the potential for the prevention of suffering and premature death.

## Abbreviations

AML: acute myeloid leukemia
FA: Fanconi anemia
GvHD: graft versus host disease
HNSCC: head and neck squamous cell carcinoma
HSCT: hematopoietic stem cell transplantation
MDS: myelodysplastic syndrome
SCC: squamous cell carcinoma
